# On clinical trial fragility due to patients lost to follow up

**DOI:** 10.1186/s12874-021-01446-z

**Published:** 2021-11-20

**Authors:** Benjamin R. Baer, Stephen E. Fremes, Mario Gaudino, Mary Charlson, Martin T. Wells

**Affiliations:** 1 Department of Statistics and Data Science, Cornell University, Ithaca, NY US; 2 Schulich Heart Centre, Sunnybrook Health Science Centre, University of Toronto, Toronto, ON Canada; 3 Department of Cardiothoracic Surgery, Weill Cornell Medicine, New York, NY US; 4 Department of Medicine, Weill Cornell Medicine, Weill Cornell Medicine, New York, NY US

**Keywords:** Fragility index, Statistical significance, Research methods, Loss to follow up, Sensitivity analysis, CABG

## Abstract

**Background:**

Clinical trials routinely have patients lost to follow up. We propose a methodology to understand their possible effect on the results of statistical tests by altering the concept of the fragility index to treat the outcomes of observed patients as fixed but incorporate the potential outcomes of patients lost to follow up as random and subject to modification.

**Methods:**

We reanalyse the statistical results of three clinical trials on coronary artery bypass grafting (CABG) to study the possible effect of patients lost to follow up on the treatment effect statistical significance. To do so, we introduce the LTFU-aware fragility indices as a measure of the robustness of a clinical trial’s statistical results with respect to patients lost to follow up.

**Results:**

The analyses illustrate that clinical trials can either be completely robust to the outcomes of patients lost to follow up, extremely sensitive to the outcomes of patients lost to follow up, or in an intermediate state. When a clinical trial is in an intermediate state, the LTFU-aware fragility indices provide an interpretable measure to quantify the degree of fragility or robustness.

**Conclusions:**

The LTFU-aware fragility indices allow researchers to rigorously explore the outcomes of patients who are lost to follow up, when their data is the appropriate kind. The LTFU-aware fragility indices are sensitivity measures in a way that the original fragility index is not.

**Supplementary Information:**

The online version contains supplementary material available at (10.1186/s12874-021-01446-z).

## Introduction

A parallel two arm randomized clinical trial enrolls consenting patients and then assigns them to one of two arms and later observes their outcomes. The time span between the arm assignment and the observation of the patient’s outcome can be large, up to several years or even several decades. Sometimes patients cannot be located to observe their outcome. In this case, patients are said to be *lost to follow up* (LTFU). A naive analysis of clinical trial data neglects patients who were lost to follow up. Regulatory agencies have provided guidance handling missing data from patients lost to follow through documents such as the ICH E9 Revision on Estimands [1] and the NAS report on missing data [2]. These guidelines acknowledge that losing patients to follow up can be unavoidable but emphasize the importance of efforts to minimize patient loss to follow up.

The validity of results from clinical trials can be considerably reduced by low rates of patient participation and high rates of patients being lost to follow up. If the patients who are lost to follow up induce an imbalance between the trial arms, the clinical trial may give biased results; similarly this may occur if the patients who are lost to follow up are missing for different reasons across arms [3, 4]. Literature surveys have reported that 60-89% of randomized trials have some missing outcome data [5]. The NAS guidance expresses that “analysts should assess the robustness of the treatment effect inferences by conducting a sensitivity analysis” [2]. Based on a sample of articles from a top medical journal, Akl et al. (2012) estimated the percentage of trials for which the relative risk would no longer be significant under a number of assumptions about the outcomes of participants lost to follow up and found that assumptions regarding outcomes of patients lost to follow up could change the interpretation of trial results [5]. In this article we investigate the sensitivity to reversal of the significance of trial results due to loss to follow up using a fragility index approach. The methods we propose are in line with Recommendation 15 in the NAS report on missing data [2] that sensitivity analyses should be part of the primary reporting of findings from clinical trials.

In clinical trials, a statistical measure called the fragility index is increasingly used as an interpretable supplement to classical measures of evidence like the *p* value [6–8]. The fragility index is defined for 2×2 contingency tables as the number of patients whose outcomes must be modified to reverse statistical significance. The fragility index measures the degree to which a clinical trial’s results depend on a few patients. For example, researchers have found that studies sometimes have a fragility index of 1, indicating that modifying the outcome of only one patient reverses statistical significance and changes the trial’s conclusion [6]. This of course indicates a problem with a significant-or-not approach to evaluating statistical hypotheses [9] but also reveals the importance of having a clinically interpretable measure for the amount of evidence against a null hypothesis.

Researchers commonly use the fragility index in part to understand the possible influence of patients who are lost to follow up [6, 10–22]. The approach taken is to compare the fragility index to the number of patients lost to follow up: when the fragility index is smaller than the number of patients lost to follow up, there is a suggestion that there is cause for concern that the patients lost to follow up could reverse statistical significance had their outcomes been available.

Both measures are patient counts, so this procedure initially seems sensible. However, the measures are fundamentally incompatible [23]. The fragility index calculation modifies patient outcomes from event to nonevent or vice versa and hence does not change the number of patients in either arm. This does not correspond to adding patients who are lost to follow up back into the trial and exploring their possible outcomes, thereby increasing the number of analyzed patients.

In this manuscript, we apply the fragility index approach to appropriately understand the impact of patients who are lost to follow up. We assume that the trial follow up happens in a way conducive for our analysis. Specifically, we assume that a dichotomous follow up measurement is made at a particular time and that the patient is not measured before that follow up time. In the “Statistical methods” section, we introduce a family of measures, called the LTFU-aware fragility indices, which adapt the fragility index to the LTFU setting. Then in the “Examples” section, we review three examples of the LTFU-aware fragility indices applied to clinical trials. In the “Discussion” section, we discuss the role of the LTFU-aware fragility indices in clinical practice. In the “Conclusion” section, we conclude the paper.

## Statistical methods

The fragility index due to Walsh et al. [6] is comprised of two main features: (1) it only considers patients for which the outcomes are known, regardless of whether they are events or nonevents, and (2) it modifies patient outcomes so that statistical significance reverses. We introduce a method tailored for understanding the effect of patients lost to follow up by relaxing the component (1) while maintaining component (2). In our view, component (2) is the core of the fragility index concept since it allows researchers to consider alternative clinical trial outcomes from the same patients. For convenience, we describe the patients with observed outcomes as the *observed patients* and the patients who are lost to follow up as the *lost patients*.

The proposed method, the *LTFU-aware fragility index*, finds the number of lost patients who must have outcomes different than expected based on the observed patients to reverse statistical significance. Calculating a LTFU-aware fragility index has two high-level steps:

1. Impute an outcome for the lost patients. Form an augmented contingency table which includes both the observed patients with observed outcomes and the lost patients with imputed outcomes.
2. Find outcome modifications which reverse statistical significance of the augmented contingency table.

Each Step will rely on a Bayesian motivated but frequentist grounded statistical methodology. Since the outcomes of the lost patients are unknown, they can sensibly be treated as random variables. The Bayesian approach we use is intuitively designed so that the best estimate of the incidence among lost patients is closely related to the incidence among observed patients. The second step will only make high posterior probability, or sufficiently likely, modifications.

### The statistical model

We now introduce some necessary notation and the statistical model. For convenience, we describe the two arms of the clinical trial as being the control and treatment arms. The notation is defined in Table 1. In the notation, the subscript denotes whether the term is for observed patients or lost patients and the superscript (when present) denotes whether the term is for the control or treatment arm. For notational simplicity, we describe the model without referring specifically to the control or treatment arm as shown in the third row of Table 1. However, the same model applies to both arms.

**Table 1:** The notation needed to set up the statistical model. The same notation holds for lost patients with an “ *ℓ*” replacing the “*o*” in the subscript

	Observed	Observed	Observed
	patient cnt	event cnt	incidence
Control	$n_{o}^{C}$	$X_{o}^{C}$	$p_{o}^{C}$
Treatment	$n_{o}^{T}$	$X_{o}^{T}$	$p_{o}^{T}$
Either	*n*_*o*_	*X*_*o*_	*p*_*o*_

The statistical model we assume is that

1 $$\begin{array}{*{20}l} X_{o} \mid p_{o} &\sim \text{Binomial} \left(n_{o}, p_{o} \right) \end{array} $$

2 $$\begin{array}{*{20}l} X_{\ell} \mid p_{\ell} &\sim \text{Binomial} \left(n_{\ell}, p_{\ell} \right)  \end{array} $$

3 $$\begin{array}{*{20}l} p_{o} & \sim \text{Beta}(1/2, 1/2) \end{array} $$

4 $$\begin{array}{*{20}l} p_{\ell} \mid p_{o} & \sim \text{Beta}(s p_{o} + 1, s - s p_{o} + 1)  \end{array} $$

for some user-supplied hyperparameter *s*>0. We also specify that each distribution is independent, within and between each arm.

The model reasonably assumes that the event counts among both the observed patients and the lost patients follow a Binomial distribution. The model also includes the well-known non-informative Jeffreys prior for the observed incidence *p*_*o*_ [24].

**Fig. 1:**
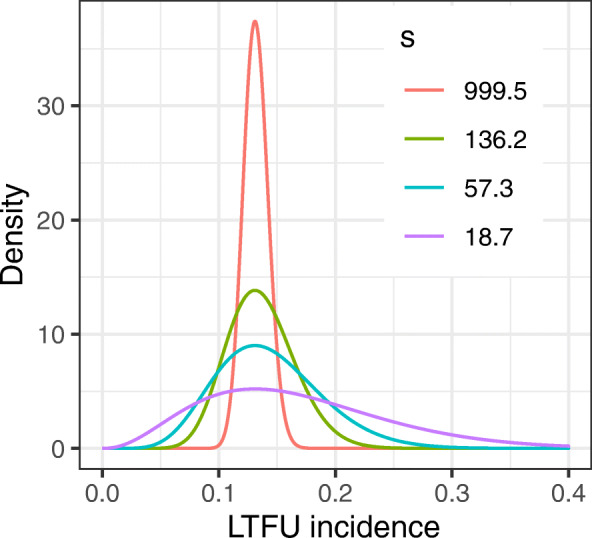
A visualization of the prior in Eq. (4) when *p*_*o*_=0.131 for various choices of *s*

The model also has the incidence *p*_*ℓ*_ among the lost patients dispersed relative to the incidence *p*_*o*_ among the observed patients. This allows for the incidences to differ between the observed patients and the lost patients. The conditional prior distribution *p*_*ℓ*_∣*p*_*o*_ is designed so that its mode is simply *p*_*o*_ [25]. The dispersion is controlled by the hyperparameter *s*. A visualization of the shape determined by various choices of *s* is in Fig. 1, where we chose *p*_*o*_=0.131 for convenience as it is the GOPCABE off-pump incidence (described in the “Examples” section). When *s*→*∞*, the probability mass of *p*_*ℓ*_∣*p*_*o*_ concentrates at *p*_*o*_, that is *p*_*ℓ*_=*p*_*o*_. This encapsulates a missing at random assumption for the lost patients [26]. When *s*=0, the observed and lost incidences are independent, encapsulating a strong not missing at random condition. This parameter can be used to reflect uncertainty about the extent to which patients are not missing at random, since in reality lost patients may be missing as they are unusual relative to the observed patients.

There are several factors which could influence the extent to which lost patients are not missing at random. We believe primary factors include the degree of sickness, the acuteness of illness, whether the trial is local or international, and the country where the trial is based. By default, we choose *s* so that a 75% equal tail probability interval has right end point which is 1.3 times higher than the sample proportion $\hat {p}_{o}$. We believe this is a neutral amount of additional uncertainty. In Fig. 1, the value of *s* was chosen for each density so that this multiplier was either 1.1,1.3,1.5 or 2 times. The green curve corresponding to *s*=136.2 is determined by the multiplier 1.3.

The statistical model has four unknown parameters: the incidences in the control arm and treatment arms for both the observed and the lost patients. For most clinical trials and for this manuscript, the null hypothesis for the statistical test is

$$H_{0}: \frac{p_{o}^{C} n_{o}^{C} + p_{\ell}^{C} n_{\ell}^{C}}{n_{o}^{C} + n_{\ell}^{C}}=\frac{p_{o}^{T} n_{o}^{T} + p_{\ell}^{T} n_{\ell}^{T}}{n_{o}^{T} + n_{\ell}^{T}},$$

i.e. that the overall incidences are identical in both arms. Note, our proposed methodology naturally extends to other hypotheses as well.

### Step 1: imputation

Equipped with the statistical model, we can now find a method to impute outcomes for the lost patients. To start, we find *X*_*ℓ*_∣*X*_*o*_ for both the control and treatment arm. This conditional distribution reflects two stages of uncertainty. First, there’s uncertainty due to not knowing the true incidence among the observed patients. Conditioning on *X*_*o*_ allows us to directly estimate the true observed incidence *p*_*o*_, yet estimates are not perfect. Second, due to Eq. (4) in the statistical model, there’s uncertainty due to not knowing the true incidence *p*_*ℓ*_ among the lost patients, even if we knew the true incidence *p*_*o*_ among the observed patients.

The conditional distribution *X*_*ℓ*_∣*X*_*o*_ is not in closed-form in general. Therefore we use a sampling algorithm for computation. Specifically, we follow a three part sampling scheme: sample from *p*_*o*_∣*X*_*o*_, sample from *p*_*ℓ*_∣*p*_*o*_, and then sample from *X*_*ℓ*_∣*p*_*ℓ*_. Note, all parts are in closed form including *p*_*o*_∣*X*_*o*_ since the Beta and Binomial distributions are conjugate. We then discard the *p*_*o*_ and *p*_*ℓ*_ samples, thus marginalizing over them.

Note, the conditional distribution is in closed-form when *s*→*∞* so that the only source of uncertainty is the first and *p*_*o*_=*p*_*ℓ*_. Then, the only source of uncertainty is due to not knowing the common expected incidence. In this case, we can view *X*_*ℓ*_∣*X*_*o*_ as a posterior predictive distribution [24], and it can be derived that *X*_*ℓ*_∣*X*_*o*_∼Beta-binomial(*n*_*ℓ*_;*X*_*o*_+0.5,*n*_*o*_−*X*_*o*_+0.5). The Beta-binomial distribution is overdispersed relative to the Binomial distribution in Eq. 2 and so reasonably models the additional uncertainty.

The conditional distribution is also in closed form when *s*=0 so that a strong form of the lost patients not missing at random is assumed. In this case, the prior distribution *p*_*ℓ*_∼Uniform[0,1] is independent of the observed incidence *p*_*o*_. The posterior distribution is $X_{\ell } \sim \text {Uniform}\{0, \dots, n_{\ell }\}$.

Next, to impute the event count among the lost patients, we determine an estimate by summarizing *X*_*ℓ*_∣*X*_*o*_. The expected value is not necessarily an integer count, so we cannot use it for imputation in general. Instead, we use the mode of the conditional distribution. We use the mode instead of the median for reasons explained in the next subsection. Note, whenever the mode is not unique, we carry out the following process simultaneously for each mode and ultimately report the median count of outcome modifications.

After the imputation, an augmented contingency table is created which has an observation for both the observed patients and the lost patients. The observed patients have unchanged outcomes but the lost patients have imputed outcomes.

### Step 2: outcome modifications

With the augmented contingency table from Step 1 completed, we now find counts of patient outcome modifications which reverse statistical significance to complete Step 2. Recall that outcome modifications are at the core of the fragility index concept.

We will consider a subset of outcome modifications when minimizing the number of outcome modifications to reverse statistical significance. The subset will be the outcome modifications which have high enough probability or are *sufficiently likely*. The LTFU-aware fragility index depends on a user-supplied number *q*, which is the probability threshold which controls the likelihood of the permitted outcome modifications [27] in Step 2. When *q* is small, any outcome modification is permitted and the LTFU-aware fragility index is the absolute minimum number of outcome modifications which reverses statistical significance. As *q* grows, the rarest outcome modifications will no longer be permitted and so reversed statistical significance must be achieved through more likely outcome modifications and hence the LTFU-aware fragility index will be larger.

Let *CI*_*q*_ be the (1−*q*)*%* posterior highest density region (HDR) for the event counts among the lost patients in both the control and treatment arms $(X_{\ell }^{C}, X_{\ell }^{T})$. The HDR *CI*_*q*_ is analogous to a frequentist confidence interval and shares many of the same theoretical properties [24]. Because we imputed the outcomes of the lost patients by using the posterior mode, we know that the imputation is in all (nonempty) highest density regions. A crucial perspective that we leverage below is that the HDR *CI*_*q*_ is a collection of the outcomes of the lost patients for which outcome modifications to them are sufficiently likely according to the probability threshold *q*.

To find the LTFU-aware fragility index for the threshold *q*, the proposed algorithm has the following steps.

- Restrict to only considering patient outcomes in the highest density region *CI*_*q*_, i.e. which are sufficiently likely, and which are associated with reversed statistical significance.
- Find the outcome which requires the fewest outcome modifications to reach from the imputation, i.e. the posterior mode.
- Return the corresponding count of outcome modifications.

When reversing significance is impossible so that the second part of Step (a) removes all outcomes, the LTFU-aware fragility index is undefined or infinite [27].

## Examples

In this section, we provide examples of the LTFU-aware fragility indices on real and simulated clinical trials. We chose each clinical trial example in order to illustrate a spectrum of fragility.

The statistical test we use for detecting a treatment effect with dichotomous data is two-sided Fisher’s exact test with significance threshold 0.05, although any test and any threshold would suffice.

### GOPCABE: a non-fragile result

The German Off-Pump Coronary Artery Bypass Grafting in Elderly Patients (GOPCABE) trial [28] was a randomized, controlled, multicenter trial conducted to investigate the benefits of coronary-artery bypass grafting (CABG) without cardiopulmonary bypass in the elderly. The study included patients who were at least 75 years of age undergoing first-time CABG. Eligible patients were randomly assigned to off-pump CABG or on-pump CABG. The primary end point was a composite of death or a major adverse event within 30 days and within 12 months after surgery. After some exclusions, 1191 patients in the off-pump arm and 1212 patients in the on-pump arm underwent CABG. After surgery, 2 patients withdrew consent and 7 patients were lost to follow up at 30 days. At 12 months, an additional 23 patients were lost to follow up and 1 patient had withdrawn consent. A total of 1179 patients assigned to off-pump CABG and 1191 patients assigned to on-pump CABG were available for analysis of the 12-month end point.

The GOPCABE trial was analyzed using a time-to-event analysis, which is the best practice when such data is available. The GOPCABE trial found an insignificant difference between the off-pump and on-pump CABG. However, for the purposes of our example, we will coerce their data to be dichotomous so that the LTFU-aware fragility index can be applied. After making the trial outcomes dichotomous (either composite event or not), Fisher’s exact test for the trial outcomes shown in Table 2 agrees with this finding and returns a *p* value of 0.509.

**Table 2:** The experimental data from the GOPCABE trial [28]

	Event	Non-event	LTFU
Off-pump	154	1025	12
On-pump	167	1024	21

**Fig. 2:**
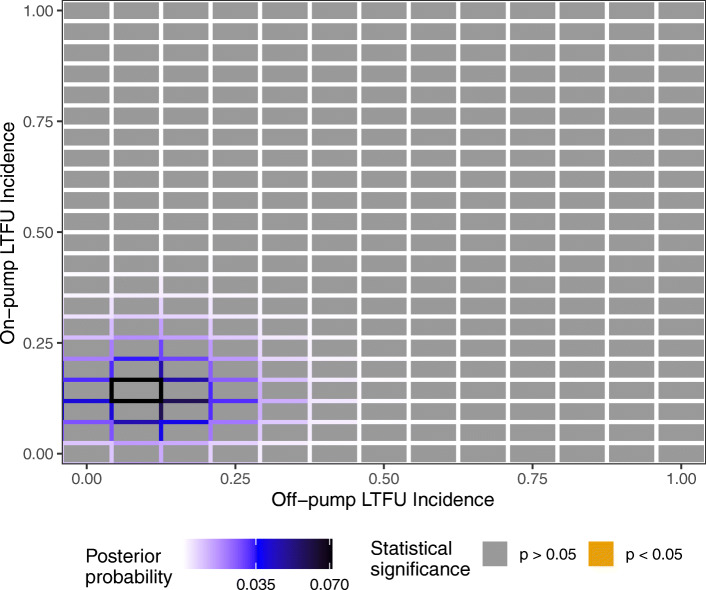
Information for each possible outcome for the lost patients in Table 2. The posterior probability is shown via the border coloring, and the statistical significance of the augmented data is shown with the tile color

We found that there’s no combination of outcomes that the lost patients could have had which reversed statistical significance, as shown in Fig. 2. The color of each tile indicates that the Fisher’s exact on the augmented contingency table is never significant. Because the LTFU count is so low, the effect sizes remain approximately constant when exploring the lost patient outcomes: in the off-pump arm, the effect size ranges from 0.129 to 0.139. Therefore, the LTFU-aware fragility index can be considered to be undefined or infinite [27]. Further, the posterior probability of the lost patients reversing statistical significance is 0. The GOPCABE trial result is not fragile once the lost patients are taken into account.

### EXCEL: moderately fragile results

The Evaluation of XIENCE versus Coronary Artery Bypass Surgery for Effectiveness of Left Main Revascularization (EXCEL) trial was an international, open-label, multicenter, randomized trial that compared PCI everolimus-eluting stents with CABG in patients with left main coronary artery disease [29]. Patients were eligible to participate in the EXCEL trial if they had various predefined stenosis of the left main coronary. The primary outcome was the composite of death from any cause, stroke, or myocardial infarction.

The EXCEL trial was primarily analyzed using a time-to-event analysis based on the restricted mean survival time [30], which found an insignificant difference between the two arms using a time-to-event analysis. However, the EXCEL trial investigators also considered a dichotomous analysis based only on the assigned arm of the study, and we focus on that approach since it allows the LTFU-aware fragility indices to be applied. Stone et al. (2019) showed that the risk of death, stroke, or MI was 22.0% in the PCI arm and 19.2% in the CABG-treated patients, a difference that was not statistically significant (*p*=0.13).

Using data in Figure S1 of the Supplementary Appendix in [29] it can be shown that there was a significant difference in the LTFU rates in the two arms (*p*=0.01). In the dichotomous analysis in Stone et al., all lost patients were assumed to not have an event. This is a problematic assumption because it is of course not likely true but also because of the discrepancy between the LTFU counts in the arms of the trial. Such an assumption makes the CABG incidence seem artificially lower due to the higher patient loss to follow up rate in the CABG arm. The EXCEL investigators addressed these problems through a sensitivity analysis involving imputation of the outcomes of the lost patients. We now expand on their sensitivity analysis.

**Fig. 3:**
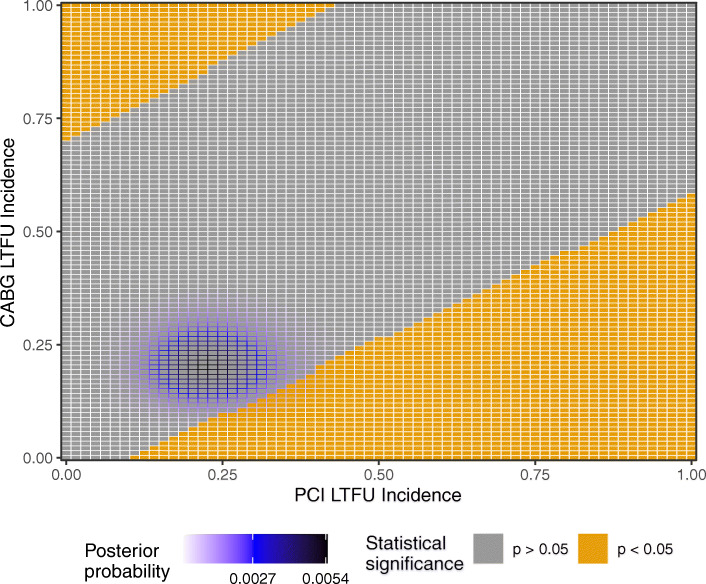
Information for each possible outcome for the lost patients in Table 3. The posterior probability is shown via the border coloring, and the statistical significance of the augmented data is shown with the tile color

In Fig. 3, we visualize the statistical significance associated with each possible outcome of the lost patients. The color of each tile indicates whether Fisher’s exact on the augmented contingency table is significant or not. At the bottom right of the plot, there are lost patient outcomes for which PCI has statistically significantly higher composite risk than CABG. At the top left of the plot, there are lost patient outcomes which establish statistical significant in the opposite direction. Already, the EXCEL trial result seems moderately fragile since both possible significance conclusions could be realized if the outcomes of the lost patients became available. Because the LTFU counts within each arm are considerable, the incidences can noticeably vary upon taking into account the lost patients. In the CABG arm, the incidences can vary from 0.184 when no lost patients have an event to 0.283 when all do.

**Table 3:** The experimental data from the EXCEL trial [29]

	Event	Non-event	LTFU
PCI	203	681	64
CABG	176	686	95

Figure 3 also visualizes a posterior distribution for the likelihood of each possible outcome. The prior distributions were chosen so that *s*=64.3 in the PCI arm and *s*=76.2 in the CABG arm, each based on the empirical incidence among the observed patients. The purple shading shows where the most likely outcomes are located. The most likely outcome, which we impute, is 14 events (i.e. 21.9% incidence) and 19 events (i.e. 20% incidence) in the PCI and CABG arms, respectively. The augmented data with these imputed outcomes is statistically insignificant. We now consider nearby alternative outcomes which produce statistical significance. When *q*=0 so that any outcome is considered sufficiently likely, a modification of only 12 lost patient outcomes would produce statistical significance and establish that CABG has lower composite risk than PCI, with effect size 0.025. The smallest credible region which contains an outcome which reverses statistical significance has *q*=0.251. When we consider only those outcomes as sufficiently likely, a modification of 13 patient outcomes is needed to similarly reverse statistical significance.

We’ve calculated the LTFU-aware fragility indices for various choices of the sufficiently likely threshold *q*. We found that around a dozen outcome modifications from the imputed most likely outcomes of the lost patients were needed to turn the statistically insignificant result into a significant result. Given the size of the EXCEL trial and the uncertainty associated with the outcomes of the lost patients, we feel that this is a notably low number. Researchers should use the LTFU-aware fragility indices reported here to contextualize the EXCEL trial’s statistical conclusions.

### CABG arterial pressure: a very fragile result

We now explore the fragility of a clinical trial studied by Peterson et al. and Gold et al. [31, 32]. The Gold et al. trial was conducted from 1991–1994 among coronary artery bypass graft surgery patients and investigated the effect of High mean arterial pressure (MAP) (treatment) versus Low MAP (control) during cardiopulmonary bypass. The event of interest was a composite of 5 complications: cardiac morbidity/mortality, neurologic morbidity/mortality, all-cause mortality, neurocognitive dysfunction, and functional decline. Peterson et al. [31] employed a routine 6 month follow up strategy.

Additionally, as we will elaborate on later in this subsection, they also conducted an extensive home follow up strategy to make non-lost many of the patients who would otherwise be lost to routine follow up [31]. Note, there were 11 patients who were altogether lost, but for illustration purposes we do not consider these patients.

The outcomes of the clinical trial are shown in Table 4. the trial had many patients lost to routine follow up: The Low MAP and the High MAP arms had 18.5*%* and 26.6*%* of enrolled patients lost, respectively. The Low MAP and High MAP incidence are 31.7*%* and 19.8*%*, respectively. The difference between expected incidences in both arms is insignificant, *p*=0.071.

**Table 4:** The experimental data from the Gold et al. clinical trial [31]

	Event	Non-event	LTFU
Low MAP	32	69	23
High MAP	18	73	33

**Fig. 4:**
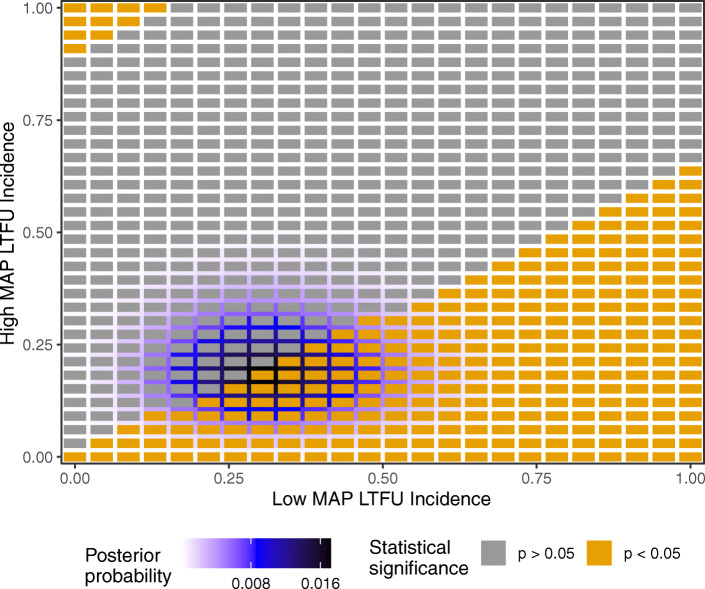
Information for each possible outcome for the lost patients in Table 4. The posterior probability is shown via the border coloring, and the statistical significance of the augmented data is shown with the tile color

In Fig. 4, all possibilities for the combinations of lost event counts are shown. As before, the color of each tile indicates whether Fisher’s exact on the augmented contingency table is significant or not.

The prior distributions were chosen so that *s*=38 in the Low MAP arm and *s*=79.7 in the High MAP arm, each based on the empirical incidence among the observed patients. The most likely outcome for the lost patients is 7 and 6 events in the Low MAP and High MAP arms, respectively. This tile has a black outline in Fig. 4. Therefore, in Step 1 of our algorithm, we impute these outcomes for the lost patients to form the augmented contingency table.

Amazingly, the augmented data has a statistically significant treatment effect with a *p* value of 0.041. Therefore, the LTFU-aware FI is 0 for any choice of the threshold *q* that permits any outcomes. The study conclusion of insignificance is so fragile that lost patients having their expected outcomes reverses statistical significance. The effect size among the observed patients is 11.9*%*; the effect size after incorporating the lost patients and reversing significance is 12.1*%*.

This clinical trial example producing a LTFU-aware fragility equal to 0 is not contrived. Whenever the difference in observed incidences is nonzero, having a large enough sample size with similar observed incidences will result in a statistically significant test. This is simply because the evidence that the expected incidences are different will grow as the number of patients with the same observed incidences grow.

The fragility of the study conclusion is further emphasized by considering the outcomes of the patients lost to routine follow up [31]. Among the 23 and 33 patients lost to routine follow up in each respective arm, they found that 2 patients in the Low MAP arm and 2 patients in the High MAP arm additionally had an event. Incorporating these outcomes of the lost patients results in a *p* value equal to 0.045, which is statistically significant.

Despite the test being statistically significant upon taking into account the lost patients as our model-based approach predicts, the actual event counts of the lost patients were lower than the imputed values 6 and 7. Indeed, having 2 events in both arms is considered so unusual by our model that the posterior credible interval needs to have at least 95.5*%* coverage to include that value. This challenges the standard intuition that adverse events are more likely among lost patients. However, note that there were still 11 patients altogether lost to follow up which were not considered in this analysis and could for instance have all had an event; if they did, the incidences would indeed be higher among the lost patients.

### Further examples

Note that alternatives plots which illustrate the effect size and alternative prior specifications on the three above trials are in the supplement. Supplementary Figs. S1, S2, and S3 study the effect size. Supplementary Figs. S4, S5, and S6 study prior specifications with the lost patient incidence biased towards zero. Supplementary Figs. S7, S8, and S9 study prior specifications with the lost patient incidence biased towards 1/2. These additional analyses are in line with Recommendation 15 in the NAS report on missing data [2] that examining sensitivity to the assumptions about the missing data mechanisms is a mandatory component of the reporting.

## Discussion

In this section, we discuss some salient aspects of the methodology. In the “Literature review” section, we discuss the relationship between the LTFU-aware fragility indices and existing work concerning lost patients. In the “The relationship between *p*_*o*_ and *p*_*ℓ*_” section, we discuss the relationship between the incidences among the observed and lost patients and its consequences for interpretation of the LTFU-aware fragility indices. In the “Methodological comments” section, we discuss methodological aspects of the LTFU-aware fragility indices.

### Literature review

The LTFU-aware fragility index is not the first approach to evaluating and understanding the impact of lost patients on a clinical trial’s statistical conclusions. In our view, earlier approaches in the literature have been more coarse due to the lack of the fragility index concept. Akl et al. (2012) [5] considered the most extreme possible outcomes for the lost patients and determined whether they would reverse the significance of a statistical test. This procedure is sometimes called a classical *tipping point analysis*. The classical tipping point analysis can roughly be viewed as determining whether LTFU-aware fragility index exceeds a certain threshold rather than finding the precise LTFU-aware fragility index.

The classical tipping point analysis has been extended to more comprehensive and flexible approaches. Ouyang et al. (2017) define an approach which imputes the outcomes of the lost patients under the missing at random assumption, then patients in the treatment arm have their outcomes modified from nonevent to event until statistical significance reverses. The percentage change from the original treatment arm incidence to the control arm incidence is defined as the sensitivity metric [33]. This approach closely parallels the LTFU-aware fragility indices, except that outcomes are modified in a single arm and a single direction and a percentage is returned. Note, the first point is reminiscent of the fragility index algorithm initially proposed by Walsh et al. (2014), which was reviewed and critiqued by Baer et al. (2021) [27].

There have also been other statistical methods developed to account for the lost patients. An epidemiological approach which closely parallels our imputation strategy involves inverse probability weighting [34]. In our setting, this method would up-weight the observed patients to (roughly) impute outcomes for the lost patients such that the lost incidence *p*_*ℓ*_ equals the observed incidence *p*_*o*_, analogous to our method when *s*→*∞*. Multiple imputations are sometimes used to address missing data in clinical trials, but it is not suitable to be combined with the sufficiently likely construction here. The sufficiently likely construction itself summarizes the posterior distribution, and we are only interested in point estimation via the LTFU-aware fragility indices.

### The relationship between *p*_*o*_ and *p*_*ℓ*_

The statistical model we considered is not the only reasonable model. We specified that the observed incidence and the lost to follow up incidence were unknown before the study but also closely related. If researchers know that the lost to follow up incidence *p*_*ℓ*_ is higher than the observed incidence *p*_*o*_ by a specified amount, then incorporating that into the model is crucial. In practice, this isn’t usually known though, so treating the lost to follow up incidence as centered on the observed incidence helps the interpretability of the LTFU-aware fragility index by providing a neutral assumption from which to measure deviations. We encourage researchers to tune the prior distribution of *p*_*ℓ*_∣*p*_*o*_ for each arm when they have strong beliefs about their relationship.

If only a small number of lost patients need to have their outcomes different than expected to reverse statistical significance, there is evidence that the statistical result is fragile. There are several possible reasons for why this could be, i.e. why the patient count in the LTFU-aware fragility index could be small. First and foremost, the modelling assumptions could hold so that the incidence *p*_*ℓ*_ of the lost patients is centered at the incidence *p*_*o*_ of the observed patients. This means for instance that lost patients would have sought medical attention in the trial if they had an adverse event. In this case, the LTFU-aware fragility indices can be interpreted similarly to the typical fragility index due to Walsh et al. Second, the modelling assumptions concerning the relationship between *p*_*ℓ*_ and *p*_*o*_ could actually not hold. In this case, a LTFU-aware fragility index should be interpreted as additionally measuring the discrepancy between the incidence among lost patients and among observed patients.

### Methodological comments

In this work, we’ve assumed that the clinical trial under study has a particular structure which is inherited by the data. First, we assumed that the outcome was dichotomous, such as event or nonevent, and that the only available attribute of the patient is the arm to which they were assigned, such as control or treatment. Second, we made assumptions about the nature of the follow up. We assumed that researchers attempted to observe each patient only once, all at roughly the same time so that there were no longitudinal measurements or other time information. This made it so that for lost patients we only knew that they were lost.

These assumptions will not hold for all clinical trials. Indeed, we hope they do not: we actively encourage researchers to learn as much as possible about lost patients. Therefore, many researchers will have to coerce their data to this format to apply the LTFU-aware fragility indices, potentially throwing away useful information. In future work we plan to expand the LTFU-aware fragility index approach to other data structures. This will allow further patient attributes to inform the imputed outcomes of the lost patients.

The data structure described above is the same that the usual fragility index due to Walsh et al. requires. Therefore the usual fragility index can be calculated whenever the LTFU-aware fragility indices can be calculated. These two fragility indices capture different concepts but in some cases can be closely related. When the loss to follow up is low, the augmented contingency table underlying the LTFU-aware fragility indices is very “close” to the fully observed contingency table, suggesting a close relationship between the two fragility indices. In this case, the LTFU-aware fragility indices are still capped to be no larger than the number of lost patients unlike the usual fragility index. When many patients are lost to follow up, the fragility indices can be considerably different.

The proposed method does not directly take into account the effect size and instead focuses on statistical significance. Readers who wish to directly incorporate the effect size into the LTFU-aware fragility indices could do so in a number of ways. First, they could use a variant of the fragility index which studies both statistical significance and clinical significance [36]. Second, they could modify Step 2 of the proposed method so that in addition to seeking reversed statistical significance, the method also seeks effect sizes to be within a given clinically meaningful region.

## Conclusion

We introduced a family of fragility indices that are tailored for discerning the potential impact of the lost patients. The usual fragility index due to Walsh et al. [6] considers modifying outcomes which were observed and does not touch the lost patients. Therefore, the fragility index due to Walsh et al. considers alternative clinical trial outcomes by essentially assuming that no patients are lost to follow up and hence the full clinical trial data is available.

Since the *p* value and the fragility index due to Walsh et al. are based on the same information, the fragility index due to Walsh et al. is a measure of evidence against a null hypothesis in the same category as a *p* value [14, 37]. The fragility index due to Walsh et al. is not a sensitivity measure, insofar as *p* values are not sensitivity measures. However, the LTFU-aware fragility index is a sensitivity measure. It leaves unchanged the outcomes of the observed patients and incorporates new information from the lost patients. Therefore, the LTFU-aware fragility index cannot be a “*p* value in sheep’s clothing” [14]. The LTFU-aware fragility index provides a way for clinicians to understand the potential impact of the lost patients, in line with regulatory guidance.

It is often reported that serious bias due to LTFU does not occur until the LTFU rate is > 20*%*, and that little bias is likely if the proportion is < 5*%* [38]. Each of our examples happen to follow this rule: GOPCABE has a 1.4*%* LTFU rate and is non-fragile, EXCEL with a 8% LTFU rate and is boderline, and Peterson et al. CABG with a 22.6*%* LTFU rate and is very fragile. We view this as strictly a coincidence. The core issue to determine fragility is whether the purple region in the Figures in “Examples” section considerably intersects the tiles with reversed significance. This involves terms such as the event rates and the loss to followup count in either arm (rather than in aggregate). In order to take these into account, we recommend researchers create visualizations like above for their study rather than relying on approximate rules of thumb.

The LTFU-aware fragility indices are efficiently implemented in an open source R package FragilityTools [39, 40]. Code to exactly reproduce the figures and examples is available in the package.

## Supplementary Information

**Additional file 1** Appendix: On Clinical Trial Fragility Due to Patients Lost to Follow Up.

## Data Availability

All data generated or analysed during this study are included in this published article in Tables 2, 3, and 4.
