# Supplementary material for: On clinical trial fragility due to patients lost to follow up
Source: BMC Med Res Methodol. 2021 Nov 20;21:254. doi: 10.1186/s12874-021-01446-z (PMC8606097; doi:10.1186/s12874-021-01446-z)
Supplement: Supplementary file 1 — Additional file 1 Appendix: On Clinical Trial Fragility Due to Patients Lost to Follow Up. [file 12874_2021_1446_MOESM1_ESM.pdf]

RESEARCH

Appendix: On Clinical Trial Fragility Due to Patients Lost to Follow Up

Benjamin R. Baer[1*], Stephen E. Fremes[2], Mario Gaudino[3], Mary Charlson[4] and Martin T. Wells[1,4]

Extended examples

In this section, we explore alternative visualizations of the three example clinical trials which result from alternative choices.

Effect size focused examples

We consider alternative visualizations of each clinical trial example in Section 3. Instead of the tile color displaying whether the augmented data (with the observed and lost patients) is statistically significant, the tile color now shows the effect size of the augmented data. The results are shown in Figure 1, Figure 2, and Figure 3.

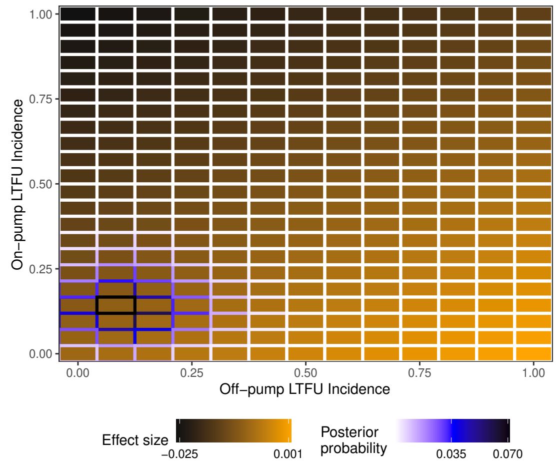

Figure 1 Information for each possible outcome for the lost patients in Table 2. The posterior probability is shown via the border coloring, and the effect size of the augmented data is shown with the tile color.

Alternative prior choices

We explore the posterior distribution of the event counts among the lost patients $X_\ell \mid X_o$ when the conditional prior distribution $p_\ell \mid p_o$ in Equation 4 is chosen to be biased towards 0 or biased towards 1/2. We will see that these biases are reflected in the posterior distribution, because the conditional prior distribution fully controls the transfer of information from the observed patients to the lost patients.

First, let us consider the former case and take

$$p_\ell \mid p_o \sim \mathrm{Beta}(sp_o/2 + 1, s(1 - p_o/2) + 1).$$

Note that this is the original definition of the conditional prior but with p_o replaced by $p_o/2$. The resulting figures for each of the three considered clinical trial examples is below. The results are shown in Figure 4, Figure 5, and Figure 6. In all cases, the the posterior distributions is biased towards 0 for both arms.

Second, let us consider the latter case and take

$$p_\ell \mid p_o \sim \mathrm{Beta}(s(p_o/2{+}1/4){+}1, s(1{-}(p_o/2{+}1/4)){+}1).$$

Note that this is the original definition of the conditional prior but with p_o replaced by $p_o/2 + 1/4$. The resulting figures for each of the three considered clinical trial examples is below. The results are shown in

*Correspondence: brb225@cornell.edu
[1] Department of Statistics and Data Science, Cornell University, Ithaca, NY, US
Full list of author information is available at the end of the article

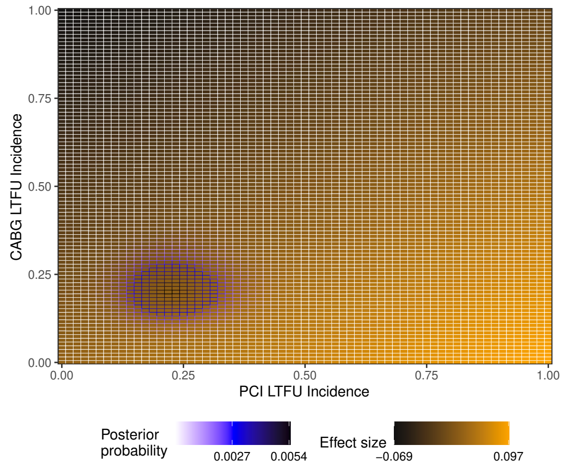

Figure 2 Information for each possible outcome for the lost patients in Table 3. The posterior probability is shown via the border coloring, and the effect size of the augmented data is shown with the tile color.

Figure 7, Figure 8, and Figure 9. In all cases, the the posterior distributions is biased towards 1/2 for both arms.

Author details

[1] Department of Statistics and Data Science, Cornell University, Ithaca, NY, US. [2] Schulich Heart Centre, Sunnybrook Health Science Centre, University of Toronto, Toronto,ON, CA. [3]Department of Cardiothoracic Surgery, Weill Cornell Medicine, New York, NY, US. [4]Department of Medicine, Weill Cornell Medicine, Weill Cornell Medicine, New York, NY, US.

References

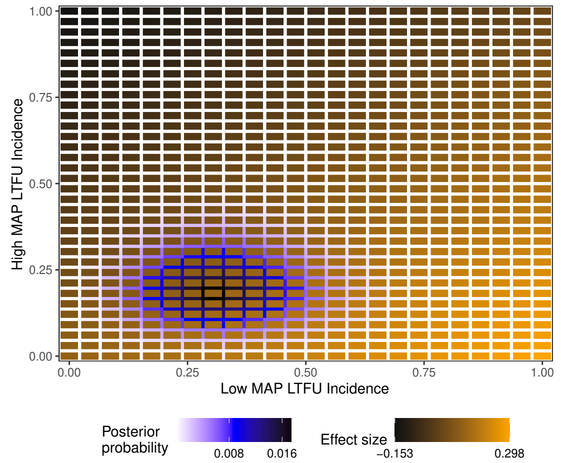

Figure 3 Information for each possible outcome for the lost patients in Table 4. The posterior probability is shown via the border coloring, and the effect size of the augmented data is shown with the tile color.

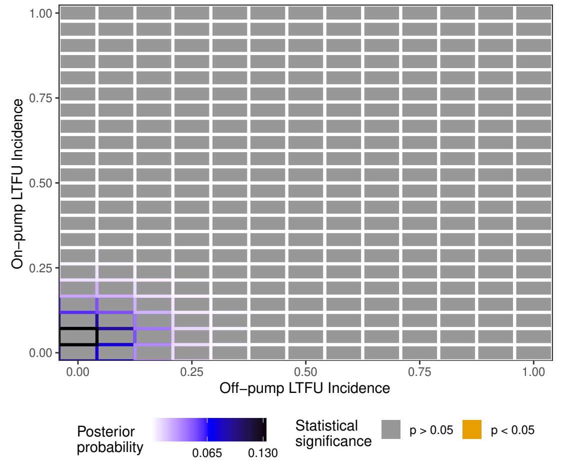

Figure 4 Information for each possible outcome for the lost patients in Table 2. The posterior probability is shown via the border coloring, and the effect size of the augmented data is shown with the tile color. The lost incidence prior is chosen to be biased towards 0.

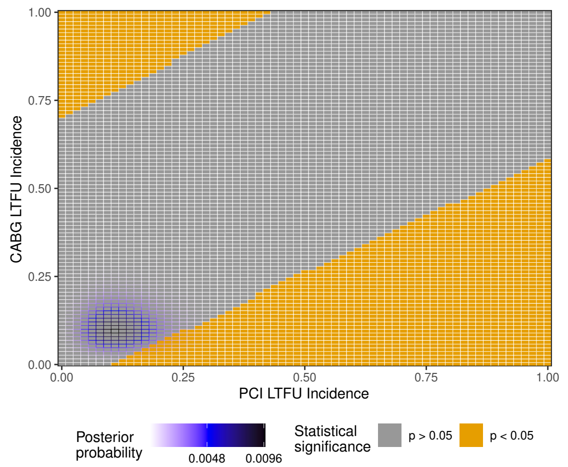

Figure 5 Information for each possible outcome for the lost patients in Table 3. The posterior probability is shown via the border coloring, and the effect size of the augmented data is shown with the tile color. The lost incidence prior is chosen to be biased towards 0.

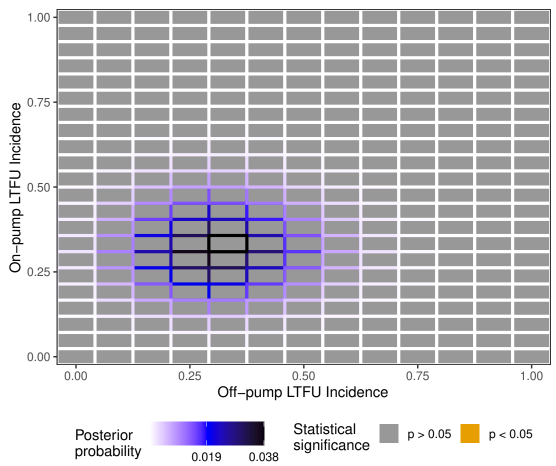

Figure 7 Information for each possible outcome for the lost patients in Table 2. The posterior probability is shown via the border coloring, and the effect size of the augmented data is shown with the tile color. The lost incidence prior is chosen to be biased towards $1/2$.

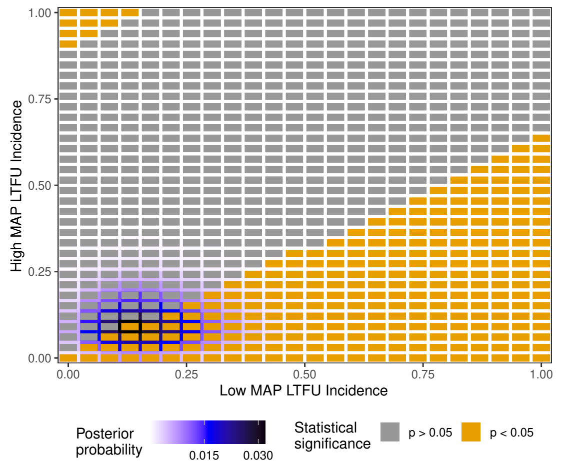

Figure 6 Information for each possible outcome for the lost patients in Table 4. The posterior probability is shown via the border coloring, and the effect size of the augmented data is shown with the tile color. The lost incidence prior is chosen to be biased towards 0.

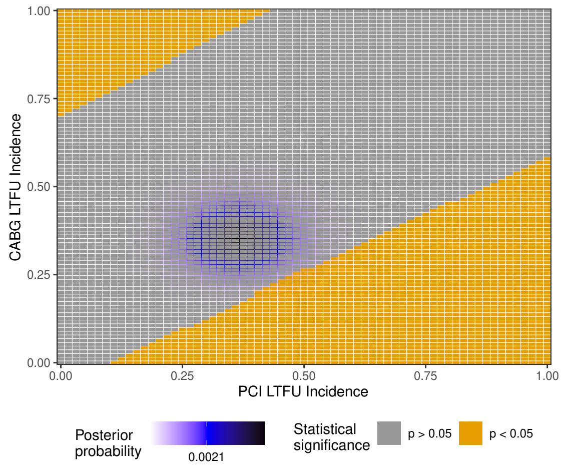

Figure 8 Information for each possible outcome for the lost patients in Table 3. The posterior probability is shown via the border coloring, and the effect size of the augmented data is shown with the tile color. The lost incidence prior is chosen to be biased towards $1/2$.

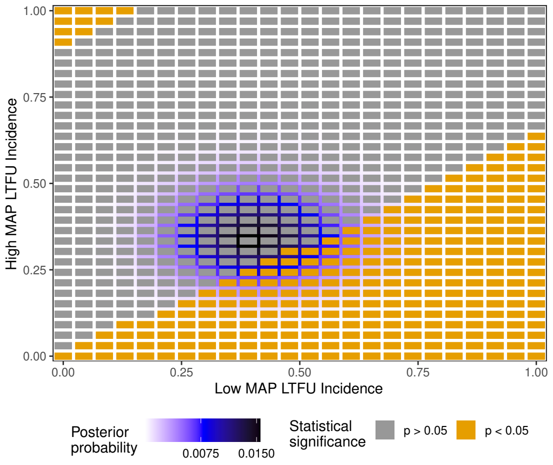

Figure 9 Information for each possible outcome for the lost patients in Table 4. The posterior probability is shown via the border coloring, and the effect size of the augmented data is shown with the tile color. The lost incidence prior is chosen to be biased towards $1/2$.
